# *c-MYC* is a radiosensitive locus in human breast cells

**DOI:** 10.1038/onc.2014.427

**Published:** 2014-12-22

**Authors:** M A Wade, N J Sunter, S E Fordham, A Long, D Masic, L J Russell, C J Harrison, V Rand, C Elstob, N Bown, D Rowe, C Lowe, G Cuthbert, S Bennett, S Crosier, C M Bacon, K Onel, K Scott, D Scott, L B Travis, F E B May, J M Allan

**Affiliations:** 1 Northern Institute for Cancer Research, Newcastle University, Newcastle upon Tyne, UK; 2 Northern Genetics Service, Institute of Genetic Medicine, Newcastle University, Newcastle upon Tyne, UK; 3 Department of Pediatrics, University of Chicago, Chicago, IL, USA; 4 Department of Biology, University of York, Heslington, York, UK; 5 Department of Histopathology, Harrogate and District NHS Foundation Trust, Harrogate District Hospital, Yorkshire, UK; 6 Department of Radiation Oncology and Rubin Center for Cancer Survivorship, James P Wilmot Cancer Center, University of Rochester Medical Center, Rochester, NY, USA

## Abstract

Ionising radiation is a potent human carcinogen. Epidemiological studies have shown that adolescent and young women are at increased risk of developing breast cancer following exposure to ionising radiation compared with older women, and that risk is dose-dependent. Although it is well understood which individuals are at risk of radiation-induced breast carcinogenesis, the molecular genetic mechanisms that underlie cell transformation are less clear. To identify genetic alterations potentially responsible for driving radiogenic breast transformation, we exposed the human breast epithelial cell line MCF-10A to fractionated doses of X-rays and examined the copy number and cytogenetic alterations. We identified numerous alterations of *c-MYC* that included high-level focal amplification associated with increased protein expression. *c-MYC* amplification was also observed in primary human mammary epithelial cells following exposure to radiation. We also demonstrate that the frequency and magnitude of *c-MYC* amplification and c-MYC protein expression is significantly higher in breast cancer with antecedent radiation exposure compared with breast cancer without a radiation aetiology. Our data also demonstrate extensive intratumor heterogeneity with respect to *c-MYC* copy number in radiogenic breast cancer, suggesting continuous evolution at this locus during disease development and progression. Taken together, these data identify *c-MYC* as a radiosensitive locus, implicating this oncogenic transcription factor in the aetiology of radiogenic breast cancer.

## Introduction

Ionising radiation is a potent human carcinogen. Several tissues and organs are susceptible to the transforming effects of ionising radiation, including the breast in adolescent and young women in whom risk is both dose- and age-dependent.^1^ In women treated with radiotherapy for Hodgkin lymphoma, there is a linear relationship between radiation exposure and breast cancer risk, with those under the age of 20 years at the time of exposure at the highest risk of developing subsequent breast cancer.^2^ Similarly, young women exposed to radiation after the atomic bombs of Hiroshima and Nagasaki^3, 4, 5^ and those exposed to high levels of diagnostic radiation^6, 7, 8, 9^ have an elevated risk of breast cancer.

The molecular genetic mechanisms that lead to radiogenic breast cancer are unclear, but there is evidence that cancers subsequent to radiation exposure have genotypic and phenotypic features that distinguish them from other breast cancers, and are more likely to be of the HER2 or basal-like subtypes.^10^ Furthermore, array comparative genomic hybridisation and expression microarray analyses demonstrate that radiogenic breast cancers cluster separately from other breast cancers^10, 11^ and have a higher degree of genetic instability, such as a higher frequency of allelic loss of chromosome bands 6q13–q14 and 9p21.^12, 13^ Specific alterations have been implicated in radiogenic breast transformation,^14, 15, 16, 17, 18^ but there is no evidence that any of these genetic changes are induced directly by radiation exposure.

Understanding the pathogenesis and underlying molecular genetic alterations that drive radiogenic breast cancer could facilitate early detection in those who have been exposed to high levels of ionising radiation and help tailor subsequent therapy. To this end, we have used human cell model systems to identify cytogenetic and copy number alterations induced by *in vitro* exposure to ionising radiation, and determined the frequency of these alterations in breast cancer patients with and without antecedent radiation exposure.

## Results

### Acquired copy number alterations in irradiated MCF-10A breast epithelial cells

**Figure 1:**
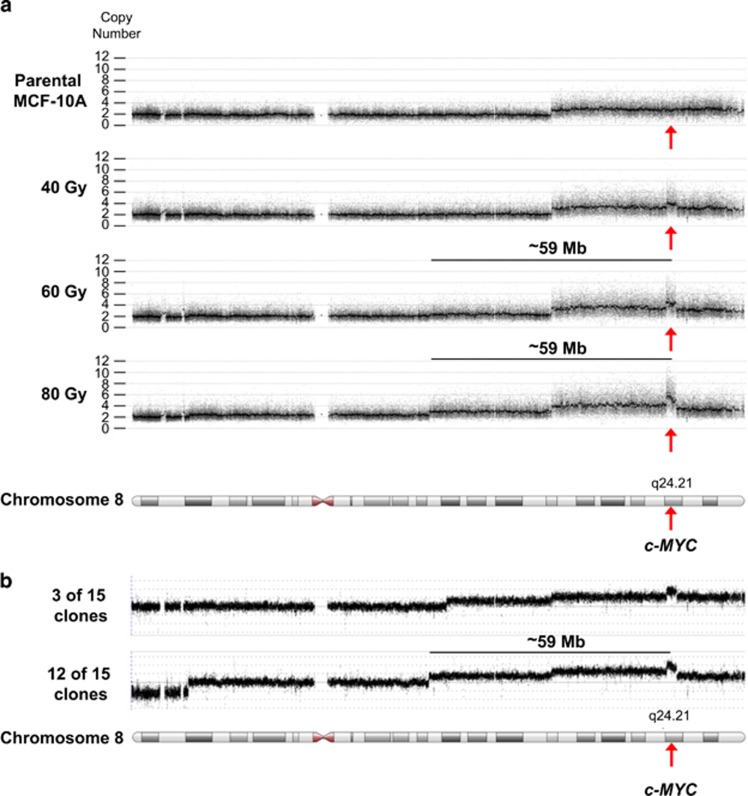
SNP6.0 and Cytoscan array copy number profile of chromosome 8 in parental and irradiated MCF-10A cell populations. Parental MCF-10A and MCF-10A cells irradiated with fractionated X-ray doses of 5 Gy to a cumulative dose of 40, 60 and 80 Gy were assessed by SNP6.0 array (**a**). Each SNP marker on chromosome 8 is represented and aligned to its position on chromosome 8 as well as its designated copy number state. An ideogram of chromosome 8 is positioned below the SNP marker plots. A 2.5 Mb copy number gain was identified in the 40 Gγ population and spans the *c-MYC* locus, increasing its copy number state from 3 to 4. The position of *c-MYC* is highlighted on each SNP marker plot and the chromosome 8 ideogram by a red arrow. An ~59 Mb copy number gain was identified in the 60 Gγ population, which spanned a number of regions with different constitutive copy number states and also encompassed the *c-MYC* locus, therefore increasing its copy number state further. The ~59 Mb copy number gain first identified in the 60 Gy population was further pronounced in the 80 Gy population. The ~59 Mb region is indicated by the horizontal black line above the SNP marker plots of the 60 and 80 Gy population. Clones from MCF-10A cells irradiated with fractionated X-ray doses of 5 Gy to a cumulative dose of 80 Gy were assessed by Cytoscan array (**b**). All 15 clones analysed carried the focal 2.5Mb amplification encompassing *c-MYC*. Twelve of the 15 clones carried the additional ~59 Mb copy number gain discernible from SNP6.0 data and a deletion affecting the p-arm of chromosome 8. Three of the 15 clones did not carry the 59 Mb copy number gain or the p-arm deletion, but carried a novel copy number gain also encompassing the *c-MYC* gene.

MCF-10A cells were irradiated with fractionated doses of X-rays to a cumulative dose of 80 Gy using four independent dosing regimens (two using 5 Gy and two using 10 Gy fractions), and copy number alterations in irradiated cells were assessed by high-density single-nucleotide polymorphism (SNP) array analysis (Affymetrix SNP6.0, Affymetrix, La Jolla, CA, USA) by comparison with parental MCF-10A. A number of large-scale and focal copy number alterations were identified (Supplementary Tables 1–3), including a 2.5 Mb focal copy number gain affecting the *c-MYC* proto-oncogene on 8q in one of the 5 Gy dosing regimens (Supplementary Table 1). Given the established role of *c-MYC* in breast cancer pathogenesis, the aetiology and evolution of this alteration was investigated in cells that had received a cumulative radiation dose of 20, 40, 60 and 80 Gy. Comparison with parental MCF-10A indicated no discernible copy number alterations in cells that had received a cumulative dose of 20 Gy. However, in the 40 Gy cumulative dose population, the 2.5 Mb copy number gain of 8q, which includes the *c-MYC, PVT1* and *TMEM75* genes, first became discernible (Figure 1a and Supplementary Table 1). This gain occurred within a 46 Mb region of chromosome 8q with an inherent copy number of 3 in parental MCF-10A.^19^

In the 60Gy cumulative dose population, a further copy number gain of ~59 Mb was apparent for chromosome 8q, which included and extended beyond the 46 Mb region of copy number gain present in parental MCF-10A (Figure 1a and Supplementary Table 1). The telomeric breakpoint of the ~59 Mb gain was unclear, but appeared to be located within the focal 2.5 Mb copy number gain encompassing *c-MYC*. The array profile of the 80 Gy cumulative dose population was broadly similar to that observed in the 60Gy population, although the large ~59 Mb copy number gain and the focal 2.5 Mb copy number gain encompassing *c-MYC* were more prominent features.

To further define the breakpoints in the proximity of the *c-MYC* locus, we isolated 15 independent cell clones from the 80 Gy population via limiting dilution and analysed these using very high-density SNP arrays (Affymetrix Cytoscan). These data identified two major sub-populations with distinct breakpoints in the proximity of the *c-MYC* locus (Figure 1b). All 15 clones carried the 2.5 Mb focal copy number gain affecting the *c-MYC* locus first identified in the 40 Gy population. Twelve of the 15 clones carried the additional ~59 Mb copy number gain discernible from SNP6.0 data. Furthermore, these additional high-density SNP data demonstrated that the telomeric breakpoint was located within *PVT1* (Supplementary Figure 1). This major clone also carried a deletion affecting the p-arm of chromosome 8 (telomere-13 726 906). Three of the 15 clones did not carry the ~59 Mb copy number gain or the p-arm deletion described above. Rather, these clones carried a novel copy number gain also encompassing *c-MYC* (75 234 254– telomere), which was not discernible from the SNP6.0 data, presumably because these cells constituted a minor sub-population. Taken together, these data suggest that these two sub-populations, with additional alterations affecting the *c-MYC* gene, share an ancestral clone carrying the focal 2.5 Mb amplification. This model demonstrates additional breaks in the proximity of *c-MYC* and ongoing evolution of this locus as a result of cumulative radiation exposure.

### *c-MYC* alterations in parental and irradiated MCF-10A breast epithelial cells

**Figure 2:**
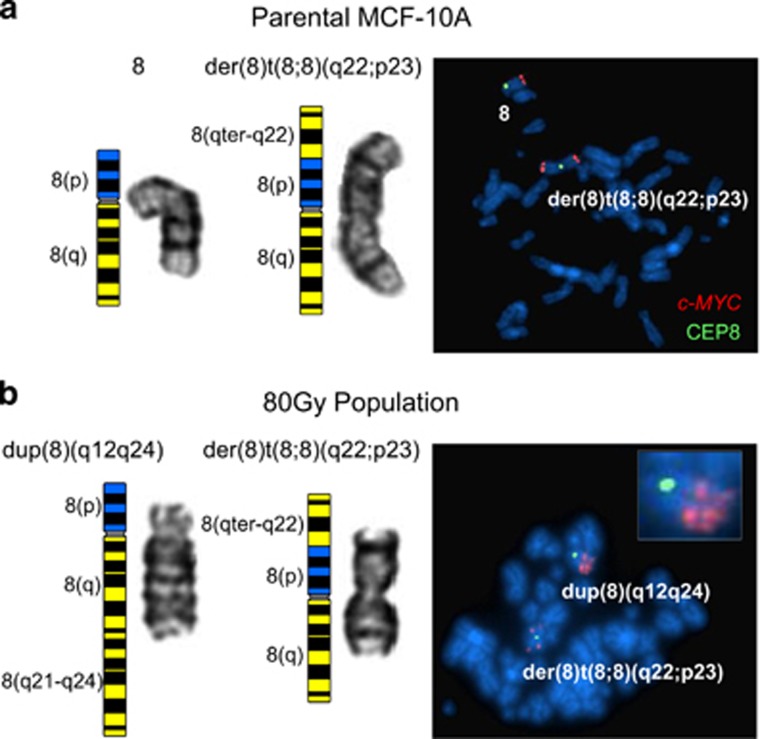
Alterations that affect *c-MYC* in MCF-10A cells. Partial karyotype analyses of chromosomes 8 and FISH analysis for *c-MYC* (red probe) and chromosome 8 centromere (green probe) on 4′,6-diamidino-2-phenylindole (DAPI) counterstained metaphase nuclei in parental MCF-10A (**a**) and 80 Gy cumulative dose cells (**b**). Chromosome arms are labelled on the ideograms adjacent to the karyotype images. The 46 Mb region of chromosome arm 8q gain present in parental MCF-10A cells is due to a duplication of 8qter-q22 and subsequent translocation to the end of the short arm resulting in a derivative chromosome 8: der(8)t(8;8)(q22;p23). The der(8)t(8;8)(q22;p23) is identified in both the parental and 80 Gy MCF-10A cell populations. The 80 Gy population has a second abnormal chromosome 8, which comprises a tandem duplication of the 8q12–q24 region to the q-telomere of the constitutively normal chromosome 8: dup(8)(q12–q24). FISH analysis confirmed that *c-MYC* is present in both the constitutive 46 Mb region of gain on der(8)t(8;8)(q22;p23) and the duplicated region on dup(8)(q12–q24) identified in the 80 Gγ population. The magnified view of the acquired dup(8)(q12–q24) chromosome in the 80 Gγ population (inset in **b**) shows that multiple copies of *c-MYC* are present at both expected 8q24 chromosome positions.

The allelic location, magnitude and orientation of the alterations affecting *c-MYC* in parental MCF-10A and cells from the 80 Gy irradiated population were assessed by cytogenetic and metaphase fluorescence *in situ* hybridisation (FISH) analysis. The region of 8q gain present in parental MCF-10A cells was shown to be a duplication and subsequent inversion to the end of chromosome 8p, which results in a derivative chromosome, der(8)t(8;8)(q22;p23) (Figure 2a).

The large ~59 Mb gain first identified in the 60 Gy population and generated via the break in *PVT1* is a tandem duplication of the 8q12–q24 region of the normal chromosome 8 giving rise to dup(8)(q12–q24) (Figure 2b). FISH identified multiple copies of *c-MYC* at both the expected 8q24 bands on the dup(8)(q12–q24) chromosome (Figure 2b, inset), demonstrating that the ~59 Mb duplication and the focal amplification are on the same allele. No evidence that the ~59 Mb duplication preceded the focal amplification was provided by metaphase FISH, and supports the SNP array data in suggesting that the *PVT1* break and ~59 Mb duplication occurred subsequent and independent to the 2.5 Mb focal amplification.

Additional alterations that affected the *c-MYC* locus were identified in minor sub-populations of cells that had received a cumulative dose of 80 Gy (Supplementary Figure 2) and included a translocation to an unidentified partner chromosome, a further duplication of the der(8)t(8;8)(q22;p23) and a second tandem duplication on the dup(8)(q12–q24).

### *c-MYC* amplification occurs at low cumulative radiation dose

**Figure 3:**
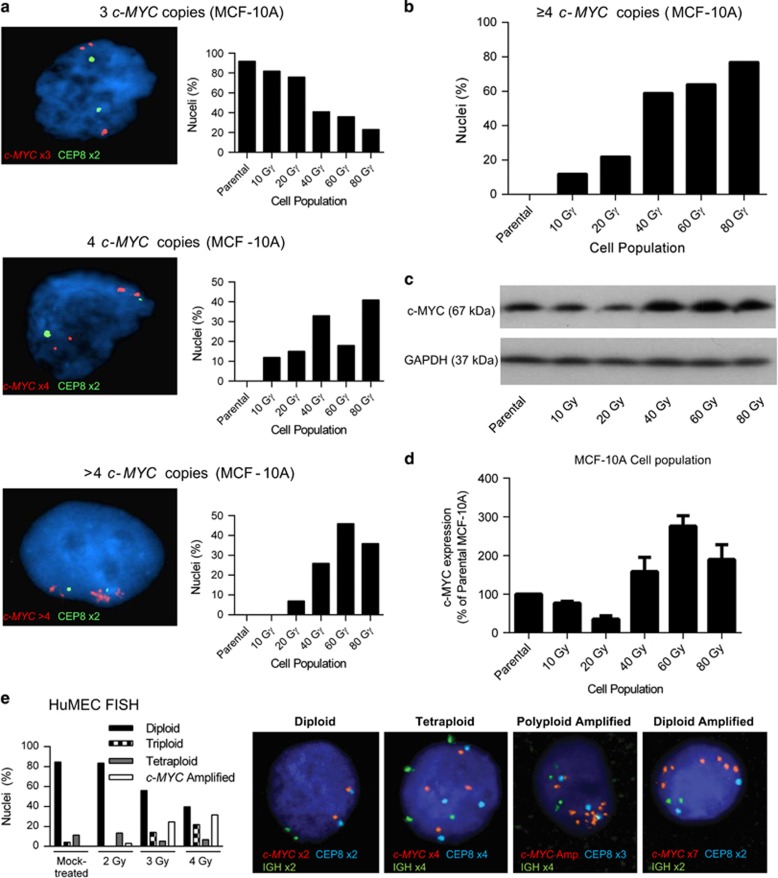
Genotypic and phenotypic alterations of *c-MYC* in irradiated huMECs and MCF-10A cells. *c-MYC* interphase FISH copy number analysis of parental and irradiated MCF-10A populations. Three main cell populations were identified by FISH: cells with two copies of chromosome 8 centromere (green probe) and three copies of *c-MYC* (red probe), cells with two copies of chromosome 8 and four copies of *c-MYC* and cells with two copies of chromosome 8 and over four copies of *c-MYC* (**a**). The proportion of 100 scored nuclei with these three *c-MYC* genotypes was combined and determined for each population. The proportion of nuclei with ⩾4 copies of *c-MYC* and therefore any cell population with a *c-MYC* copy number gain was determined (**b**). Aliquots of 10 μg of protein extracted from parental and irradiated MCF-10A cell populations were electrophoresed on polyacrylamide gels and analysed for c-MYC and glyceraldehyde 3-phosphate dehydrogenase (GAPDH) expression by western transfer analysis as described in the Materials and methods (**c**). c-MYC expression was quantified for each cell population by densitometric analysis of western blots from three independent protein samples (**d**). c-MYC expression is expressed as a percentage of c-MYC expression in parental MCF-10A, which is set at 100% expression. Expression of c-MYC was significantly higher in the 60 Gy population than parental MCF-10A (Turkey's test; *P*<0.05). *c-MYC* interphase FISH copy number analysis of parental and irradiated HuMECs (**e**). Four main cell populations were identified by FISH: diploid cells (two copies of chromosome 8 centromere (aqua probe), two copies of IGH (green probe) and two copies of *c-MYC* (red probe)); triploid cells (three copies of each locus); tetraploid cells (four copies of each locus); cells with amplification of *c-MYC*. At least 70 interphase cells were counted at each radiation dose (mock-treated, 2, 3 and 4 Gy) and example images are shown for diploid, tetraploid and *c-MYC-*amplified cells.

*c-MYC* alterations were analysed by FISH in 100 interphase nuclei from parental MCF-10A cells and irradiated cell populations. Three predominant *c-MYC* genotypes were identified: three *c-MYC* and two centromere 8 FISH signals (the parental MCF-10A genotype); four *c-MYC* and two centromere signals and more than four *c-MYC* and two centromere 8 signals (Figure 3a). It was not possible to discern consistently the exact *c-MYC* copy number because of signal clustering. Nevertheless, up to 12 discrete *c-MYC* hybridisation signals were apparent in some cells (Figure 3a).

Nuclei with four or more *c-MYC* hybridisation signals were not detected in parental MCF-10A cells (Figure 3a). Nuclei with four and more than four *c-MYC* FISH signals were identified first in the 10 and 20 Gy populations, respectively, which suggests that copy number gain of *c-MYC* occurred earlier in this irradiation series than was apparent from SNP array analysis (Figure 3b).

The dominance of *c-MYC*-amplified cells in the 80 Gy population suggests positive selection by sequential exposure to ionising radiation. Consistent with this model, *c-MYC*-amplified cells were significantly more resistant to the cytotoxic effects of ionising radiation compared with parental non-amplified cells (two-way analysis of variance, *P*=0.0027; Supplementary Figure 3), although the phenotype was relatively modest (parental MCF-10A IC_50_ (half-maximal inhibitory concentration)=3.1 Gy; c-MYC-amplified 80 Gy MCF-10A IC_50_=4.4 Gy).

### Copy number gain of *c-MYC* results in increased c-MYC protein expression

Concomitant with *c-MYC* amplification, c-MYC protein expression was higher in cells irradiated with a cumulative dose of 40 Gy or greater compared with parental cells (one-way analysis of variance, *P*<0.001) (Figures 3c and d). These data demonstrate that low doses of ionising radiation induce copy number alterations in human MCF-10A cells, and that *c-MYC* is susceptible to multiple structurally diverse alterations, including high-level amplification, which results in increased expression of c-MYC protein.

### *c-MYC* alterations in primary HuMECs

To exclude the possibility that radiation-induced *c-MYC* copy number gain was specific to immortalised cells, we next treated low passage human mammary epithelial cells (HuMECs) with ionising radiation and determined *c-MYC* copy number using FISH. Cells were treated with an initial dose of 2 Gy and subsequently treated with an additional dose of either 1 or 2 Gy, giving total cumulative doses of 2, 3 and 4 Gy. Using FISH probes for *c-MYC*, centromere 8 and *IGH*, we found evidence of polyploidy in all cell populations, including mock-treated control HuMECs. Interphase FISH provided no evidence for specific *c-MYC* amplification in mock-treated HuMECs (Figure 3e). However, there was radiation dose-dependent *c-MYC* amplification in radiation-treated HuMECs (Figure 3e). For example, in the 4 Gy population over 30% of the scored cells (*n*=73) had copy number gains of *c-MYC* relative to the control loci (centromere 8 and *IGH*). Furthermore, we found examples of diploid cells with at least seven distinct signals for *c-MYC* and other cells, with clear clustering of *c-MYC* signals (Figure 3e) indicative of focal amplification and consistent with the observations seen in MCF-10A.

### *c-MYC* alterations in radiogenic breast cancer after Hodgkin lymphoma

We investigated *c-MYC* copy number alterations and protein expression in breast cancer following radiotherapy for Hodgkin lymphoma (‘radiogenic breast cancer (RAD)', *n*=18; Supplementary Table 4) and age-matched cases for which there was no evidence of radiation exposure aetiology (‘sporadic breast cancer (SPO)', *n*=33; Supplementary Table 5).

**Figure 4:**
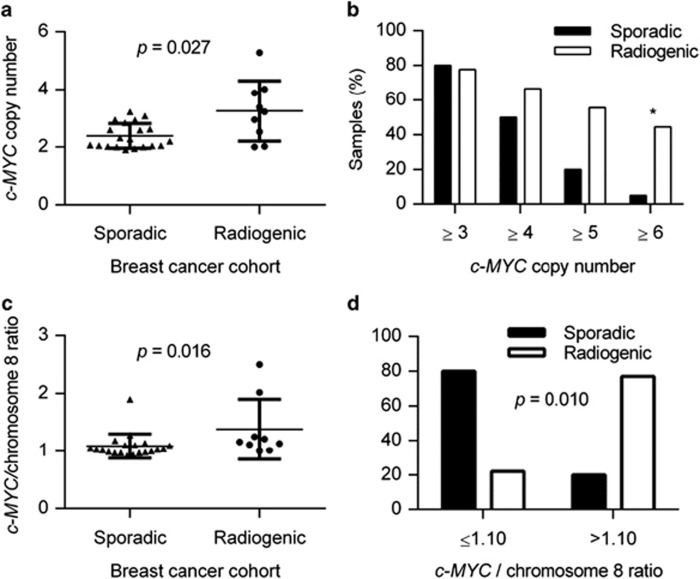
FISH analysis of *c-MYC* copy number and chromosome 8 copy number in sporadic and radiogenic breast cancers. *c-MYC* copy number was assessed in tumour cells from radiogenic cancers (*n*=9) and from sporadic cancers (*n*=20). *c-MYC* copy number was higher in the radiogenic cancers (Mann–Whitney *U*-test; *P*=0.027) (**a**). The wider horizontal bars represent the median *c-MYC* copy number and the narrower horizontal bars represent the 10th and 90th percentiles of the data. The percentage of samples in which at least 10% of the nuclei contained ⩾3, 4, 5 and 6 copies of *c-MYC* was compared (**b**) and was significantly higher in the radiogenic cohort than in the sporadic cohort for ⩾6 copies of *c-MYC* (Fisher's exact test; *P*=0.022 (*)). The ratio between *c-MYC* and chromosome 8 centromere copy number was higher in the radiogenic cohort than in the sporadic cohort (Mann–Whitney *U*-test; *P*=0.016) (**c**). The percentage of samples in each cohort, which had a *c-MYC* to chromosome 8 centromere ratio ⩽1.10 (no or little evidence of *c-MYC* amplification) or >1.10 (evidence of *c-MYC* amplification) was also compared (**d**) (Fisher's exact test; *P*=0.010).

*c-MYC* copy number was assessed by FISH and was successful for 20 of 33 (61%) sporadic breast cancer samples and 9 of 18 (50%) radiogenic breast cancer samples. *c-MYC* copy number was higher in radiogenic compared with sporadic breast cancer (Mann–Whitney *U*-test, *P*=0.027; Figure 4a; Supplementary Tables 6 and 7). Furthermore, 4 of 9 (44%) radiogenic breast cancer cases had at least 10% of nuclei with 6 or more *c-MYC* signals, compared with only 1 of 20 (5%) sporadic breast cancer cases (Fisher's exact test, *P*=0.022; Figure 4b).

### Mechanisms and magnitude of c-MYC copy number gains in radiogenic breast cancer

The pattern of centromere 8 and *c-MYC* hybridisation was studied in each sample to investigate the mechanisms that underlie the increase in *c-MYC* copy number. Although the majority of nuclei in all the samples were diploid for chromosome 8, most samples also had nuclei with more than two copies of centromere 8 (Supplementary Table 6). In some cases (e.g. SPO11 and SPO17), there were nuclei with over six centromere 8 signals. Likewise, the majority of samples had nuclei with apparent monosomy 8. These data demonstrate a high level of both intra- and intertumour heterogeneity with respect to chromosome 8 copy number status. We determined the ratio between mean *c-MYC* and chromosome 8 signals to identify cases with specific *c-MYC* locus amplification. There was a higher *c-MYC* to centromere 8 ratio in radiogenic breast cancer compared with sporadic breast cancer (Mann–Whitney *U*-test, *P*=0.016) (Figure 4c). Furthermore, more radiogenic breast cancers (7 of 9, 77%) than sporadic breast cancers (4 of 20, 20%) had a *c-MYC* to centromere 8 ratio >1.10 (Fisher's exact test, *P*=0.010; Figure 4d), indicating that locus-specific *c-MYC* copy number gain is more common in radiogenic breast cancer.

**Figure 5:**
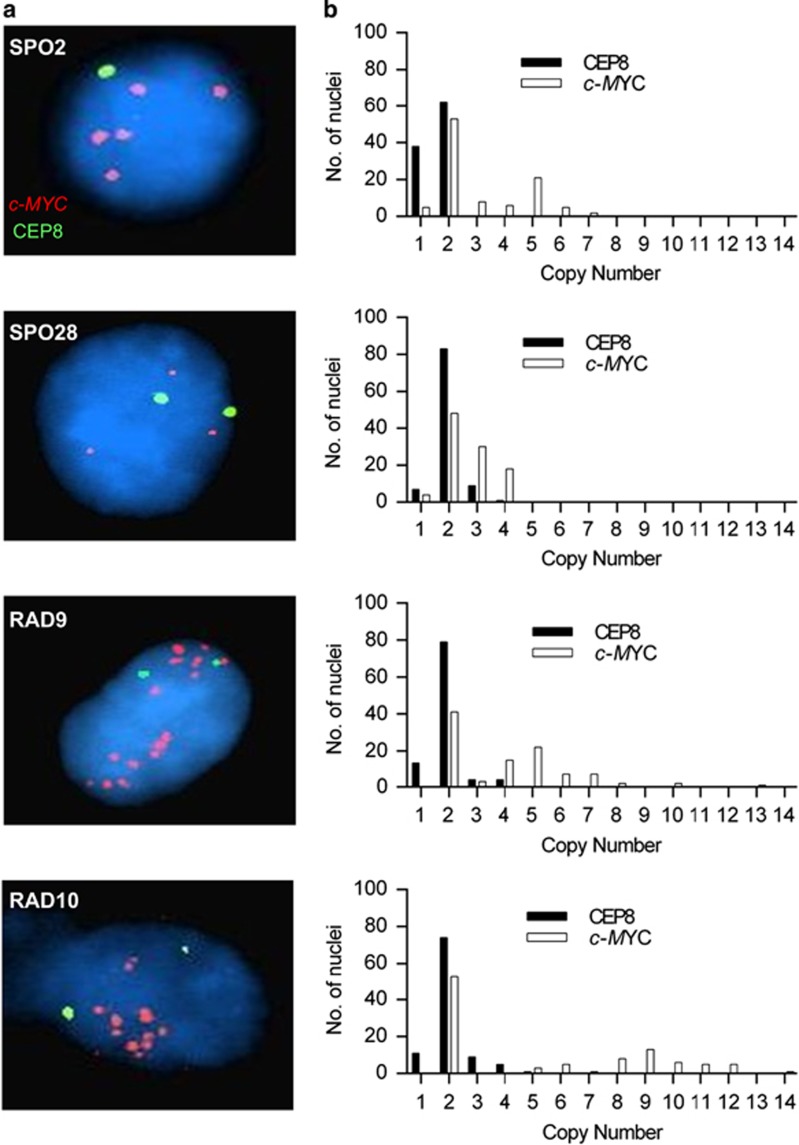
Dual-stain FISH analysis of samples that had a *c-MYC* to chromosome 8 centromere ratio >1.25. Representative FISH images of samples SPO2 (ratio=1.90), SPO28 (ratio=1.28), RAD9 (ratio=2.02) and RAD 10 (ratio=2.50) for combined *c-MYC* (red) and chromosome 8 centromere (green) hybridisation (**a**). The results show that *c-MYC*-amplified cell populations from the radiogenic cohort have a higher degree of amplification compared with amplified cell populations from the sporadic cohort. The heterogeneity of *c-MYC* and chromosome 8 centromere copy number status is shown in the histograms to the right of the images for each sample (**b**).

Two sporadic (e.g. SPO2, SPO28) and two radiogenic cases (e.g. RAD9, RAD10) had high *c-MYC* to centromere 8 ratios (>1.25) with evidence of high-level *c-MYC* locus amplification in the majority of nuclei (Supplementary Tables 6 and 7). Dual hybridisation analysis of these four *c-MYC*-amplified cases demonstrated that the magnitude of *c-MYC* copy number gain was higher in the radiogenic breast cancers compared with the sporadic cancers (Figure 5). Specifically, for SPO2 and SPO28, the majority of nuclei with *c-MYC* amplification had five and three *c-MYC* signals, respectively, and included cells with up to seven and four *c-MYC* signals, respectively (Figure 5b and Supplementary Table 6). For RAD9 and RAD10, the majority of nuclei with *c-MYC* amplification had 5 and 9 *c-MYC* FISH signals, respectively, and included cells with up to 13 and 14 *c-MYC* signals, respectively (Figure 5b and Supplementary Table 6).

Taken together, these data indicate that *c-MYC* copy number gain is more common in radiogenic breast cancer than in sporadic disease, and that a greater proportion of radiogenic samples have *c-MYC* copy number gains that affect specifically the *c-MYC* locus.

### c-MYC expression in radiogenic breast cancer and correlation with c-MYC copy number

**Figure 6:**
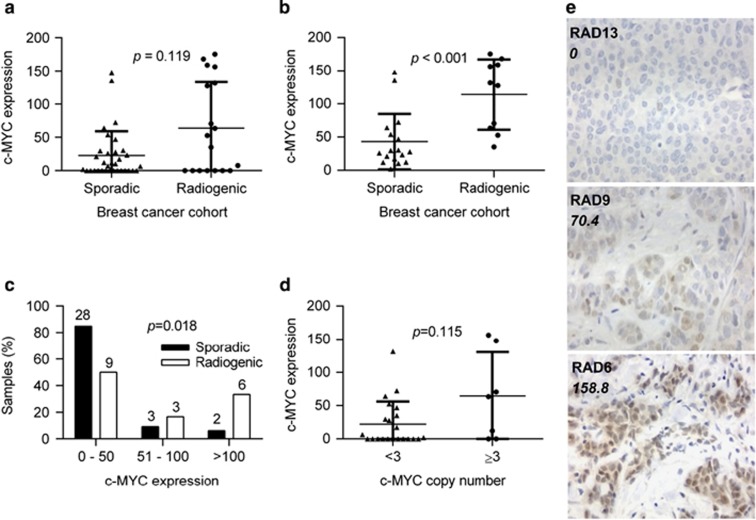
c-MYC protein expression in radiogenic and sporadic breast cancers. Formalin-fixed, paraffin-embedded tissue from sporadic (*n*=33) and radiogenic (*n*=18) breast cancers were sectioned and analysed by immunohistochemistry with a specific c-MYC antibody as described in the Materials and methods. The level of c-MYC expression was quantified by derivation of a histoscore. c-MYC expression in the sporadic and radiogenic breast cancers was compared for all cases (**a**) and was higher in radiogenic cases in which more than 10% of the nuclei had detectable c-MYC expression (Mann–Whitney *U*-test: *P*<0.001) (**b**). The proportion of sporadic and radiogenic breast cancer that had c-MYC expression histoscores of 0–50, 51–100 and >100 were compared (*χ*^2^: *P*=0.018) (**c**). The number of samples in each group in panel (**c**) is identified above each bar of the histogram. c-MYC expression is shown in tumours known to have a *c-MYC* copy number of <3 (*n*=22) or ⩾3 (*n*=7) (**d**). The wider horizontal bars represent the median *c-MYC* copy numbers and the narrower horizontal bars represent the 10th and 90th percentiles of the data (**a**, **b** and **d**). Representative IHC images of samples with no c-MYC expression (RAD13: histoscore=0), moderate expression (RAD9: histoscore=70.4) and high expression (RAD6; histoscore=158.8) are shown in (**e**).

We determined c-MYC protein expression in radiogenic and sporadic breast cancers by immunohistochemistry and derivation of a histoscore (Supplementary Figure 4). There was a trend towards higher expression of c-MYC in the radiogenic breast cancer compared with sporadic breast cancer (Figure 6a; Mann–Whitney *U*-test, *P*=0.119). Tumours in which c-MYC immunoreactivity was detected in <10% of cells were considered to be negative for expression (8 radiogenic (44%) and 16 sporadic breast cancer cases (48%)). An analysis of all cases suggested that exposure to ionising radiation was not associated with c-MYC-positivity (*χ*^2^=0.076: *P*=0.782). However, comparison of protein expression in c-MYC-positive tumours demonstrated that the expression was significantly higher in the radiogenic breast cancer compared with sporadic breast cancer (Figure 6b; Mann–Whitney *U*-test: *P*<0.001).

Samples were divided into three groups on the basis of c-MYC protein expression: no or low c-MYC expression (histoscore: 0–50), moderate expression (histoscore: 51–100) and high expression (histoscore: >100). The proportion of samples with no or low, moderate or high c-MYC expression were different between the radiogenic and sporadic cases, with high c-MYC expression significantly more common in the radiogenic series (Figure 6c; *χ*^2^=8.041; *P*=0.018).

We investigated if there was a correlation between *c-MYC* gene copy number and protein expression and if there was a trend towards higher c-MYC expression in samples with a mean *c-MYC* copy number ⩾3 (*n*=7) compared with samples with a *c-MYC* copy number <3 (*n*=22) (Mann–Whitney *U*-test, *P*=0.115; Figure 6d), although there was inconsistency between *c-MYC* copy number and c-MYC protein expression in several cases (Supplementary Table 8). In summary, these data demonstrate that c-MYC expression is higher in radiogenic breast cancer compared with sporadic disease. In some tumours, high *c-MYC* copy number is reflected in high c-MYC protein expression but in others the additional copies of *c-MYC* do not result in high levels of protein expression.

## Discussion

We identified ionising radiation-induced genetic alterations affecting *c-MYC* in immortalised non-transformed breast epithelial cells that include high-level focal amplification, duplication and translocation. A particular strength of our study is that *c-MYC* amplification was generated directly by ionising radiation in a controlled experimental system. Previous studies have suggested that *c-MYC* amplification is a late-stage event in radiation-induced transformation.^20, 21^ However, detailed single-cell analysis in this study has identified several structurally diverse alterations of *c-MYC* after relatively low radiation doses of 10–20 Gy. We also identified high-level *c-MYC* amplification in primary mammary epithelial cells following radiation doses between 2 and 4 Gy.

High-level *c-MYC* amplification was more common in human breast cancer, which developed after radiotherapy compared with breast cancer without antecedent radiation exposure, providing additional evidence that ionising radiation specifically induces *c-MYC* amplification and implicates radiotherapy as one cause of 8q alterations in radiogenic breast cancer. In support of this supposition, recurrent amplification of the *c-MYC* locus has been reported in breast cancers that developed in atomic bomb survivors.^14^

Detailed single-cell analysis also revealed considerable intra- and intertumour heterogeneity with respect to *c-MYC* copy number in human radiogenic breast cancer, suggesting continuous evolution at this locus during disease development and progression. Taken together, these observations suggest progressive accumulation of alterations and provide strong evidence that this locus is particularly sensitive to radiation-induced alteration.

There is evidence suggesting that *c-MYC* is intrinsically unstable in human breast cells. Kadota *et al.*^22^ identified a spontaneous single-copy *c-MYC* gain in MCF-10A cells, but there was no evidence of high-level amplification. The breakpoints of the single-copy gain do not match those reported in our study, which are associated with high-level focal *c-MYC* amplification. Diverse breakpoints suggest several putative fragile sites at the *c-MYC* locus. Common fragile sites with a particular propensity to undergo breakage during replication stress exist in the human genome.^23^ Two such common fragile sites, FRA8C and FRA8D, flank *c-MYC* and breakage at these sites have been identified in cervical cancer and Burkitt lymphoma.^24^ Furthermore, radiation-induced genotoxic stress, which in turn induces replication stress, has been shown to induce breaks at fragile sites in the rat genome.^25^

Our study has clearly demonstrated an association between antecedent radiation exposure and elevated expression of c-MYC in breast cancer. A clear link between radiation-induced *c-MYC* amplification and elevated c-MYC protein expression was demonstrated *in vitro* using cultured human breast cells. This association was also seen in breast cancer tissue, although the correlation was relatively weak. Reports of a weak correlation between *c-MYC* copy number and protein expression in cancer are common, and have been described in breast, pancreatic, bladder and colon disease.^26, 27, 28, 29^ Expression of c-MYC is regulated by multiple pathways and is known to be highly regulated by cell cycle state (quiescence/proliferation), irrespective of whether there is gene amplification.^30, 31^ As such, cases in which c-MYC protein expression does not increase with gene copy number could indicate a low proliferative index or mechanisms disrupting transcription/protein stability.

Furthermore, a mechanism has been identified that could be responsible for increased c-MYC expression in radiogenic breast cancer without the requirement of *c-MYC* amplification. Best *et al.*^32^ identified a risk haplotype for radiogenic breast cancer at the *PRDM1* (*BLIMP1*) locus on 6q. PRDM1 is a negative transcriptional regulator of *c-MYC* and the risk haplotype is associated with reduced PRDM1 expression, attenuated upregulation of PRDM1 and increased expression of c-MYC in response to ionising radiation. The findings of our study and the identification of the *PRDM1* risk haplotype strongly suggest an important role for c-MYC overexpression in radiogenic breast cancer development, and we can speculate that radiation-induced *c-MYC* amplification may be an important driver of c-MYC dysregulation in some, but not all, cases of radiogenic breast cancer.

*c-MYC* is a well-established proto-oncogene, which when overexpressed drives cell proliferation,^30, 33, 34^ blocks cell differentiation,^34, 35^ promotes angiogenesis^36^ and genetic instability.^37, 38^ Overexpression of c-MYC has been associated with radiation resistance, which could confer a survival advantage during a fractioned therapeutic dosing regimen and could be an important phenotype required for cell transformation.^39, 40, 41, 42^ The application of targeted therapy against cells with dysregulated c-MYC expression^43^ could therefore prove efficacious in the treatment of radiogenic breast cancer and might have value to prophylactically inhibit the outgrowth of c-MYC deregulated cells following radiation exposure.

In conclusion, this study demonstrates that ionising radiation directly induces genetic alterations affecting *c-MYC*, including translocation and high-level focal amplification, and identifies *c-MYC* as a radiosensitive locus. Importantly, c-MYC protein expression was significantly elevated in radiation-exposed breast cells, emphasising the importance of this critical transcription factor in radiogenic breast cancer. Our findings provide insight into the aetiology of radiogenic breast cancer and may have relevance to other cancers with a radiation exposure aetiology.

## Materials and methods

### Primary mammary epithelial cells (HuMECs) and MCF-10A cells

HuMECs were obtained from Gibco (Life Technologies, Paisley, UK) and maintained in HuMEC Ready Medium (Life Technologies). MCF-10A cells were obtained from the American Type Culture Collection (ATCC, Manassas, VA, USA) and cultured in the medium as described previously^44^ supplemented with 10 nM 17-β oestradiol. MCF-10A cells are a non-transformed breast epithelial cell line derived from non-malignant human fibroblastic mammary tissue immortalised by extended cultivation in low calcium concentrations.^45^ The karyotype of MCF-10A is near diploid with a t(3;9)(p13;p22) and an unbalanced translocation between part of chromosome 5 to the short arm of the derivative chromosome 9, which results in a deletion of *CDKN2A/CDKN2B*, which is thought be responsible for the immortalisation of this cell line. MCF-10A carries a duplication of part of the long arm of chromosome 8 (8q), trisomy for chromosome 20, trisomy for the long arm of chromosome 1 (1q) and an additional copy of chr1: 198 136 766-qter (19).

### Irradiation of MCF-10A and HuMECs

MCF-10A cells were exposed to 5 and 10 Gy fractionated doses of X-irradiation (2.5 Gy/min) to a cumulative dose of 80 Gy using a D3300 X-ray system (Gulmay Medical Ltd, Chertsey, UK). To test the stability of MCF-10A cells in continuous culture, unirradiated cells were cultured under the same conditions and for the same length of time as irradiated cells (termed ‘untreated MCF-10A'). Clonal populations of cells that had received a cumulative dose of 80 Gy were generated by plating cells at a density of 1 cell per well in a 96-well plate. HuMECs were exposed to an initial 2 Gy dose of X-irradiation (2.5 Gy/min) followed by an additional dose of either 1 or 2 Gy.

### Clonogenic assay

Parental MCF-10A cells and cells from the 80 Gy population were plated at a density of 200 cells per well in a 6-well plate and irradiated (0–15 Gy) 24 h later. Cell colonies were allowed to grow for 7 days before fixation with 3:1 methanol:acetic acid and staining with crystal violet. Colonies were counted by two independent researchers from duplicate wells in three independent experiments.

### Genome-wide SNP array analysis

Genomic DNA was extracted with a QIAmp DNA Mini Kit (Qiagen, Limburg, The Netherlands) and analysed by Affymetrix Human SNP6.0 or Cytoscan Array (Affymetrix). Analysis of untreated MCF-10A cells identified acquired trisomy 7, 8, 11, 13 and 19, in addition to the trisomy 20 identified previously in parental MCF-10A cells.^20^ There was no evidence of any other acquired copy number alterations in the genome of untreated MCF-10A cells, other than those identified in parental MCF-10A cells.

### G-band karyotyping of MCF-10A cells

Metaphase chromosome spreads were prepared by incubating proliferating cells with 100 ng/ml colcemid for 4 h followed by resuspension in 75 mm KCl for 7 min. Cells were fixed by resuspension in 3:1 methanol:acetic acid before karyotyping and FISH analysis. Slide-fixed cells were incubated overnight at 60 °C and G-banded by soaking in the trypsin solution (1.9 mg/ml trypsin, 74 mm NaCl, 0.469 mg/ml NaH_2_PO_4_ and 0.937 mg/ml Na_2_HPO_4_) for 10 s and in the staining solution (8 ml Giemsa stain, 0.5 ml Leishman stain and 40ml Gurr buffer (0.469 mg/ml NaH_2_PO_4_ and 0.937 mg/ml Na_2_HPO_4_)) for 3 min. Twenty metaphase chromosome spreads were analysed for each population and the karyotypes recorded.

### FISH analysis of HuMECs and MCF-10A cells

*c-MYC* and centromere 8 copy number status of MCF-10A cell populations was determined by dual hybridisation with a Vysis LSI SpectrumOrange *c-MYC* Probe (Abbott Molecular, Maidenhead, UK; cat. no.: 05J545-011) and a CEP8 SpectrumGreen Probe (Abbott Molecular; cat. no.: 06J37-018) as recommended by the suppliers. *c-MYC* (orange), centromere 8 (aqua) and *IGH* (green) copy number status of HuMECs was determined using the Abbott IGH/MYC/CEP8 Tri-colour Dual Fusion Translocation Probe (Abbott Molecular). Slides were heated to 72 °C for 5 min and then incubated for 24 h at 37 °C in a humidified hybridisation chamber (HYBrite; Abbott Molecular). After hybridisation, slides were counterstained with 4′,6-diamidino-2-phenylindole (Vector Laboratories, Peterborough, UK). FISH was scored with an Olympus BX-61 fluorescence microscope (Olympus, Southend-on-Sea, UK) with a x100 oil objective. Images were analysed using the CytoVision 7.2 SPOT counting system (Leica Microsystems, Gateshead, UK). A minimum of 100 (for MCF-10A) or 70 (for HuMECs) nuclei were scored per test by two independent analysts.

### Western transfer analysis

Proliferating MCF-10A cells were lysed in 50 mm Tris-HCl (pH 7.5), 150 mm NaCl, 1 mm EDTA, 1% (w/v) NP-40, 0.25% (w/v) sodium deoxycholate, 1 μg/ml pepstatin, 1 μg/ml aprotinin, 1 μg/ml leupeptin, 2 mm sodium orthovanadate, 2 mm sodium fluoride and 2 mm phenylmethylsulphonyl fluoride. Aliquots of 10μg protein were separated by sodium dodecyl sulphate–polyacrylamide gel electrophoresis, transferred to nitrocellulose and incubated with antibodies specific to c-MYC (Santa Cruz Biotechnology, Santa Cruz, CA, USA; no. 262) and glyceraldehyde 3-phosphate dehydrogenase (Santa Cruz Biotechnology; FL-335). The signal was detected with SuperSignal (Thermo Scientific, Rockford, IL, USA) and exposure to X-ray film (Fujifilm, Tokyo, Japan) and quantified using Lab Works 4.0 software (Ultra-Violet Products, Upland, CA, USA). Variations in the amount of protein loaded for each sample was normalised using glyceraldehyde 3-phosphate dehydrogenase. Protein in irradiated cells is expressed relative to parental MCF-10A from three independent samples.

### Breast cancer cases

This study was approved by the United Kingdom National Research Ethics Committee (nos 06/Q1108/91 and 07/Q0904/25). Breast cancer tissue was obtained from 18 women who had received radiotherapy to the chest for Hodgkin lymphoma (termed ‘radiogenic breast cancer' samples). An age- and tumour-type matched series of breast cancer cases was selected from patients with no history of prior cancer or therapeutic radiation exposure (termed ‘sporadic breast cancer' samples).

### *c-MYC* and centromere 8 FISH of breast cancer tissue samples

Formalin-fixed, paraffin-embedded breast tissue was dewaxed, rehydrated and digested by incubation in 0.005% proteinase K 0.05 m Tris-HCl (pH 7.5), 0.01 m EDTA and 0.01 m NaCl for 30 min at 37 °C and then resuspended in 40% (v/v) methanol in phosphate-buffered saline. Samples were disaggregated and cytospin slides of isolated nuclei were generated. Samples were incubated in 100 μg/ml RNAse, 0.015 m Na_3_C_6_H_5_O_7_, 0.15 m NaCl for 30 min at 37 °C in a humidified hybridisation chamber and then dehydrated in ethanol. Nuclei were digested in 1 mg/ml pepsin, 0.01 m HCl for 30 min at 37 °C in a humidified hybridisation chamber and then washed, dehydrated and air-dried. The Vysis LSI SpectrumOrange *c-MYC* Probe (Abbott Molecular) and CEP8 SpectrumGreen Probe (Abbott Molecular) were each hybridised separately to isolated nuclei because dual hybridisation did not produce strong enough signal for several samples. FISH protocols were the same as described above except that the samples were incubated at 85 °C for 30 min and then hybridised for 48 h at 37 °C. FISH was successful for 20 of 33 (61%) sporadic breast cancer samples and 9 of 18 (50%) radiogenic breast cancer samples and 100 nuclei were scored for each probe by two independent analysts.

### Immunohistochemistry

Formalin-fixed, paraffin-embedded breast tissue was dewaxed, rehydrated and antigen retrieval was achieved by incubation in 10 mm Tris, 1 mm EDTA buffer (pH 9) for 30 s at 125 °C in a Menapath Antigen Access Retrieval Unit (A Menarini Diagnostics, Wokingham, UK). The tissue was incubated in 3% (v/v) hydrogen peroxide solution for 10 min at room temperature and then washed in 0.02 m Tris-HCl, 0.14 m NaCl and 0.1% (v/v) Tween-20. It was also incubated at room temperature for 60 min with an antibody specific for c-MYC (Epitomics, Burlingame, CA, USA; cat. no.:1472-1) in Tris-buffered saline (pH 7.6) and then with Menapath HRP Polymer (A Menarini Diagnostics) for 30 min. Sections were visualised with 3,3′ diaminobezidine and were counterstained with Gills II haematoxylin. c-MYC expression was quantified by the analysis of 100 malignant epithelial cells in each of five fields. The intensity of the immunoreaction was scored from 0 to 3 (Supplementary Figure 4) by two independent researchers. A histoscore was calculated for each case from the sum of the scores for 500 cells.
