# Supplementary material for: c-MYC is a radiosensitive locus in human breast cells
Source: Oncogene. 2014 Dec 22;34(38):4985–94. doi: 10.1038/onc.2014.427 (PMC4391966; doi:10.1038/onc.2014.427)
Supplement: Supplementary Information [file onc2014427x1.pdf]

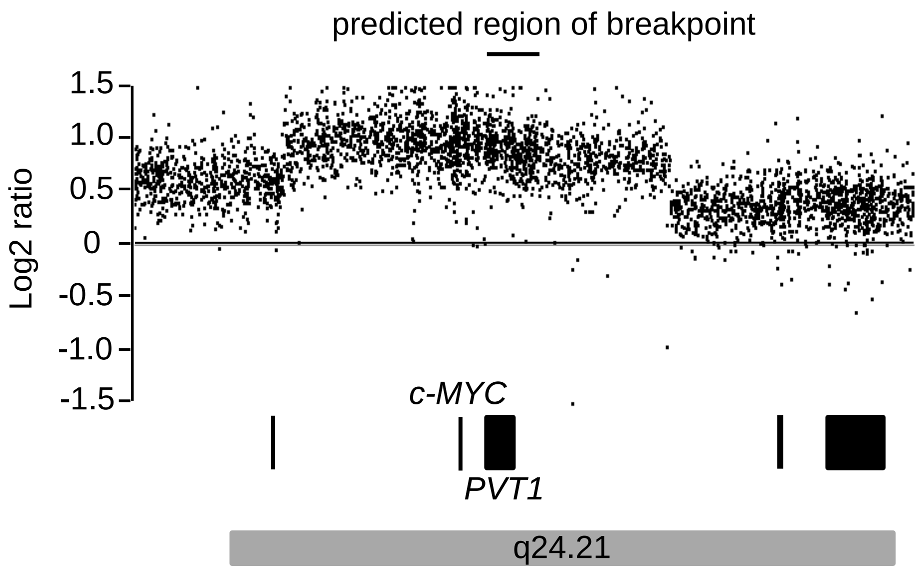

Supplementary Figure 1. Cytoscan array copy number profile of chromosome 8 q24.21 in irradiated MCF-10A 80Gy cell clone.

Cell clones derived by limiting dilution from MCF-10A cells irradiated with fractionated X-ray doses of 5Gy to a cumulative dose of 80Gy were assessed by Cytoscan array. All clones derived from the 80Gy population carried the 2.5Mb focal amplification encompassing *c-MYC*. Twelve of the 15 clones, including the one illustrated, carried the additional ~59Mb copy number gain with the telomeric breakpoint predicted in the region of the *PVT1* gene. Each point represents a single marker and their position is shown relative to cytogenetic band q24.21 and genes in this region. A log2 ratio of zero equates to a copy number of two.

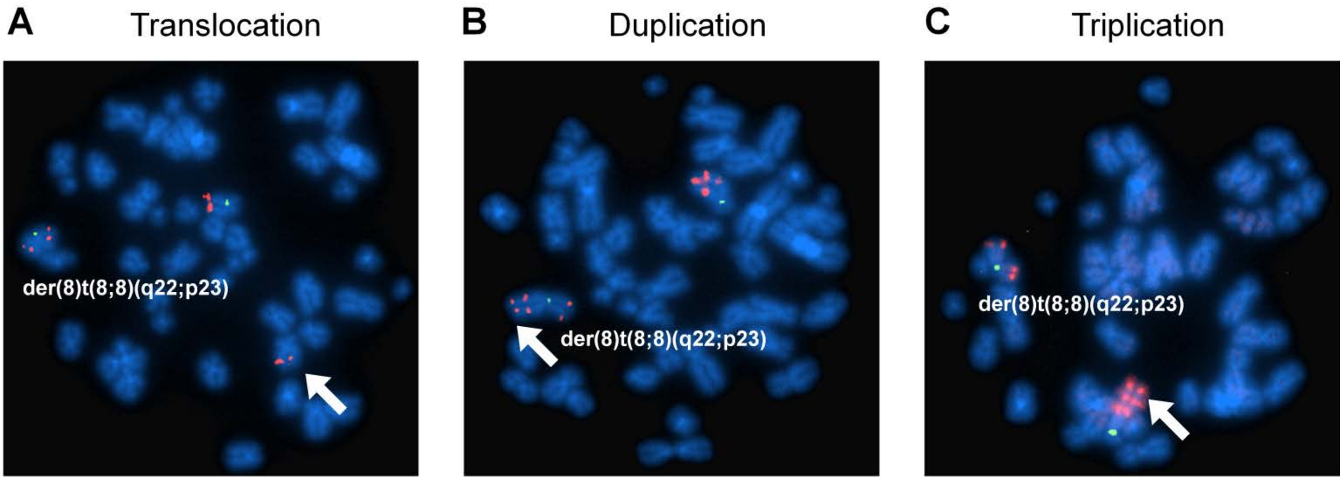

Supplementary Figure 2. Alterations affecting the *c-MYC* locus in irradiated cell clones.

Alterations affecting the *c-MYC* locus in addition to the dup8(q12-q24) and focal *c-MYC* amplification were detected by metaphase FISH analysis of the 80Gγ cell population, and included: translocation of the *c-MYC* locus to an unidentified partner locus (A); a tandem duplication on der(8)t(8;8)(q22;p23), although it was not discernible on which chromosome arm the duplication occurred (B); a second tandem duplication of the dup(8)(q12-q24) chromosome, generating an overall triplication of the *c-MYC* loci (C). Each alteration is highlighted on the FISH images by a white arrow.

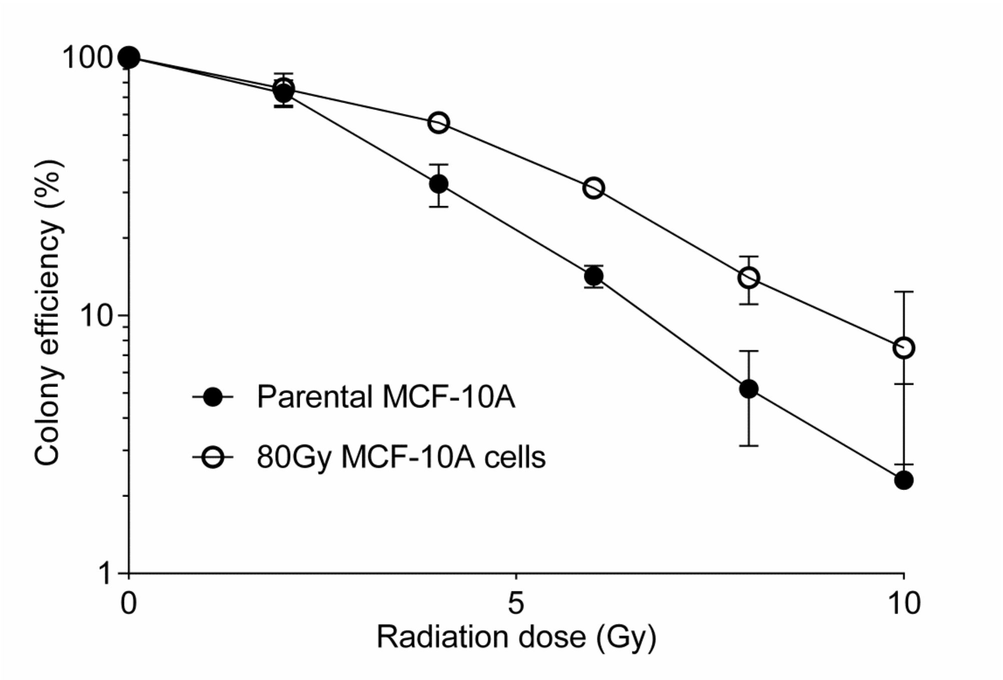

Supplementary Figure 3. Clonogenic survival of parental and 80Gy MCF-10A cell populations in response to X-irradiation.

Cell survival of parental and 80Gy MCF10-A cell populations in response to X-irradiation was determined using clonogenic survival 7 days after treatment. Plot shows mean and standard deviation from three independent experiments scored by two independent researchers. p=0.0027, two-way ANOVA

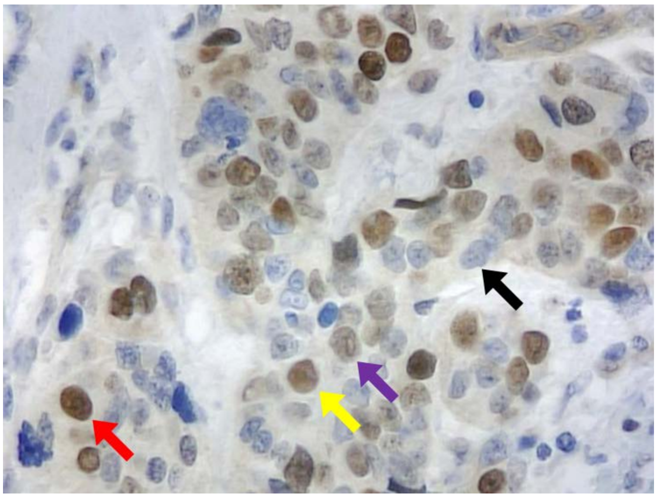

Supplementary Figure 4. Analysis of *c-MYC* expression.

Formalin-fixed, paraffin-embedded tissue from sporadic (n=33) and radiogenic (n=18) breast cancers were sectioned and analysed by immunohistochemistry with a specific c-MYC antibody as described in the Materials and Methods. Five hundred nuclei from malignant sections were assessed for c-MYC staining and assigned a score between 0 and 3 depending on the intensity of staining. The black arrow highlights an example of a nucleus assigned with a score of 0 (no c-MYC staining), the purple arrow a nucleus assigned a score of 1, the yellow arrow a nucleus assigned a score of 2 and the red arrow a nucleus assigned a score of 3. A total histoscore for each sample was calculated by the sum of the scores given to each nucleus.

Supplementary Table 1

Copy number alterations in MCF-10A cells irradiated with 5Gy fractions to a cumulative dose of 80Gy which display *c-MYC* copy number gain

Chromosome	Start Pos (bp)[A]	End Pos (bp)[A]	Size (kb)	Genes Affected	Parental MCF-10A copy number	Population alteration first detected	Nature of alteration
10q23.2	88501217	88649027	147.81	BMPR1A	2	40 Gγ	Mono-allelic deletion
8q24.21	127621008	130125337	2504.33	c-MYC, PVT1, TMEM75	3	40 Gγ	Undefined copy number gain
8q13.3-q24.21[B]	71160976	127621008 - 130125337	~ 59000	Many	2/3	60 Gγ	Mono-allelic gain

[A] Base positions represent individual SNP marker positions

[B] Represents a single alteration that spans two regions with different copy number states. Note that this alteration also spans the 2.5 Mb copy number increase that spans *c-MYC* first identified in the 40 Gγ population.

Supplementary Table 2

Copy number alterations in MCF-10A cells irradiated with 10Gy fractions to a cumulative dose of 80Gy

Acquired copy number alterations were observed in only one of the two 10Gy 10Gy irradiated MCF-10A series.

Chromosome	Start Pos (bp)[A]	End Pos (bp)[A]	Size (kb)	Genes Affected	Parental MCF-10A copy number	Population alteration first detected	Nature of alteration
2q33.3	206560040	206699229	139.189	FLJ20309, NDUFS1	2	80 Gy	Mono-allelic deletion
4p16.3-p16.1	56707	6573579	6516.872	Many	2	40 Gy	Mono-allelic deletion
9p24.3-p21.3[B]	36587	36549683	36513.096	Many	2/1	40 Gy	Mono-allelic gain
10p14	8802113	9179270	377.157	0	2	80 Gy	Mono-allelic gain
10p11.23	28428290	28483377	55.087	MPP7	2	40 Gy	Mono-allelic deletion
12q24.31-q24.33	120606484	132287718	11681.234	Many	2	80 Gy	Mono-allelic deletion
18q23.3	75798195	76009174	210.979	PQLC1, TXNL4A, C18orf22, ADNP2	2	80 Gy	Mono-allelic gain
20	Whole Chromosome				2	10 Gy	Mono-allelic gain
22q13.2	39829375	39905513	76.138	EP300	2	10 Gy	Mono-allelic deletion
Xq22.3	105900223	106395258	495.035	TBC1D8B, CLDN2, MORC4, RBM41, NUP6CL2	2	80 Gy	Mono-allelic deletion
Xq23.3	114276787	114389568	112.781	LRCH2	2	80 Gy	Mono-allelic deletion

[A] Base positions represent individual SNP marker positions

[B] Represents a single alteration that spans a number of regions regions with different copy number states.

Supplementary Table 3

Copy number alterations in MCF-10A cells irradiated with 5Gy fractions to a cumulative dose of 80Gy (second series)

Chromosome	Start Pos (bp)[A]	End Pos (bp)[A]	Size (kb)	Genes Affected	Parental MCF-10A copy number	Population alteration first detected	Nature of alteration
1q24.1-24.2	165154658	165756926	602.268	*MAEL, GPA33, DUSP27, POU2F1, CD247*	3	40 Gγ	Mono-allelic deletion
1q25.2-31.3	176867954	193499072	16631.118	Many	3	40 Gγ	Mono-allelic deletion
1q21.1-q31.1[B]	143183331	193499072	50315.741	Many	3	80 Gγ	Mono-allelic deletion
2p23.1-22.3	31176346	36986182	5809.836	Many	2	40 Gγ	Mono-allelic deletion
2q31.1	176169514	176771255	601.741	*KIAA1715, EVX2, HOXD1,2,4,8-16*	2	55 Gγ	Mono-allelic deletion
4p16.3-914	56707	37078497	37021.79	Many	2	40 Gγ	Mono-allelic deletion
5q15-q35.3[B]	93621043	180652396	87031.353	Many	2/3	40 Gγ	Mono-allelic gain
5q23.3-35.3	129632118	180652396	51020.278	Many	3	55 Gγ	Mono-allelic deletion
5q22.2	112200585	112380304	179.719	*SRP19, REEP5, DCP2*	2	80 Gγ	Mono-allelic gain
7q21.13	88668206	88771110	102.904	*ZNF8048*	2	40 Gγ	Mono-allelic gain
10p15.1	5536697	5692243	155.546	*CALML3*	2	55 Gγ	Mono-allelic gain
11q22.3-q23.3	103769002	116689124	12920.122	Many	2	40 Gγ	Mono-allelic deletion
11q12.3-q25[B]	62318238	134449982	72131.744	Many	2	55 Gγ	Mono-allelic gain
12q21.1	70892777	72832799	1940.022	*TRHDE*	2	55 Gγ	Mono-allelic deletion
14q22.3	54914289	55027066	112.777	*KIAA0831, TBPL2*	2	40 Gγ	Mono-allelic gain
15q24.2-q26.3[B]	74222695	100276767	26054.072	Many	2/1	80 Gγ	Mono-allelic gain
16p13.3	765	1985736	1984.971	Many	2	80 Gγ	Mono-allelic deletion
18q21.2-q22.3	48080079	70114155	22034.076	Many	2	40 Gγ	Mono-allelic deletion
19p13.3	41898	4609892	4567.994	Many	2	80 Gγ	Mono-allelic deletion
20p13	9293	4428933	4419.64	Many	2	80 Gγ	Mono-allelic deletion
22q13.2	39820139	39905513	85.374	*EP300*	2	10 Gγ	Mono-allelic deletion
Xp22.2	10392905	10625012	232.107	*MID1*	2	40 Gγ	Mono-allelic gain

[A] Base positions represent individual SNP marker positions

[B] Represents a single alteration that spans a number of regions regions with different copy number states.

Supplementary Table 4

Radiogenic breast cancer cases

Sample	Age of HL[A] Diagnosis (years)	Age of BC[B] Diagnosis (years)	Latency Between HL and BC (years)	Breast Cancer Pathology
RAD1	22	40	17	IDC[C]
RAD2	23	46	22	ILC[D]
RAD3	17	30	12	ILC
RAD4	19	31	11	IDC
RAD5	28	39	10	IDC
RAD6	16	39	22	IDC
RAD7	18	28	10	IDC
RAD8	22	47	24	IDC
RAD9	29	46	17	adenocarcinoma IDC
RAD10	26	45	18	IDC
RAD11	27	35	7	comedocarcinoma
RAD12	21	37	12	IDC
RAD13	29	42	13	IDC
RAD14	21	35	14	IDC
RAD15	18	41	22	IDC
RAD16	21	34	13	IDC
RAD17	16	35	19	tubular adenocarcinoma
RAD18	19	32	13	Comedocarcinoma

[A] Hodgkin Lymphoma, [B] Breast Cancer, [C] Infiltrating ductal carcinoma, [D] Infiltrating lobular carcinoma

Supplementary Table 5

Sporadic breast cancer cases

Sample	Age of BC Diagnosis (years)	Breast Cancer Pathology
SPO1	43	IDC
SPO2	43	IDC
SPO3	36	comedocarcinoma
SPO4	44	IDC
SPO5	39	IDC + DCIS
SPO6	46	IDC
SPO7	42	IDC+DCIS
SPO8	44	IDC
SPO9	38	IDC
SPO10	37	IDC + comedocarcinoma
SPO11	44	IDC
SPO12	47	IDC
SPO13	35	IDC
SPO14	47	IDC
SPO15	43	IDC
SPO16	38	IDC
SPO17	37	IDC
SPO18	41	ILC
SPO19	47	IDC + DCIS
SPO20	32	comedocarcinoma
SPO21	49	IDC
SPO22	47	IDC
SPO23	48	IDC
SPO24	33	ILC + IDC
SPO25	32	IDC + DCIS
SPO26	41	IDC
SPO27	34	IDC
SPO28	45	IDC
SPO29	37	IDC
SPO30	39	IDC
SPO31	28	mammary Paget's disease + DCIS[A]
SPO32	39	IDC + mammary Paget's disease + DCIS
SPO33	45	IDC

[A] Ductal carcinoma in situ

Supplementary Table 6

Copy number analysis of *c-MYC* and chromosome 8 in sporadic and radiogenic breast cancer.

	Copy number of probe[A]													
	1	2	3	4	5	6	7	8	9	10	11	12	13	14
SPO2	5	53	8	6	21	5	2							
	38	62												
SPO5	7	82	10	1										
	7	79	9	5										
SPO6	16	77	4	3										
	17	79	3	1										
SPO7	8	44	13	29	3	3								
	8	48	12	25	3	2	1			1				
SPO10	7	88	3	1		1								
	7	80	9	1	1	1	1							
SPO11		62	5	5	14	9	2	2	1					
		66	7	11	8	6				1	1			
SPO13	16	74		10										
	4	88	6	2										
SPO15	10	89	1											
	9	88	3											
SPO17	4	56	10	14	8	3	5							
	3	49	8	19	13	4	3	1						
SPO18	10	66	18	6										
	7	66	21	6										
SPO21	10	62	12	4	5	4	1	2						
	7	62	23	7		1								
SPO22	15	76	4	4	1									
	17	76	5	2										
SPO24	5	70	15	8	2									
	14	71	13	2										
SPO26	9	39	36	16										
	4	50	43	2	1									
SPO27	8	67	20	1	2	2								
	13	71	12	3		1								
SPO28	4	48	30	18										
	7	83	9	1										
SPO29	5	38	15	42										
	6	44	7	43										
SPO30	15	73	9	3										
	40	52	6	2										
SPO31	12	76	10	1			1							
	7	77	14	2										
SPO32	7	83	6	4										
	17	67	14	2										
RAD1		44	8	32	2	11	2	1						
	7	44	16	18	8	4	3							
RAD2	4	92	2	2										
	9	84	4	3										
RAD3	1	48	48	3										
	17	47	36											
RAD4	5	92	1	2										
	4	94	2											
RAD5		55	4	32	9									
	2	63	25	9	1									
RAD8	1	22	11	40	17	5	1	1	1	1				
	9	35	9	40	2	2	2	1						
RAD9		41	3	15	22	7	7	2		2			1	
	13	79	4	4										
RAD10		53			3	5	1	8	13	6	5	5		1
	11	74	9	5	1									
RAD17	2	56	4	6	20	12								
	2	60	5	14	18			1						

Number of nuclei					
	0-5	6-20	21-50	51-75	75-100
	0-5	6-20	21-50	51-75	75-100

[A] The copy number of each probe is indicated at the top of the table. For each sample the number of nuclei which contain a particular copy number for *c-MYC* (red) and chromosome 8 centromere (green) are shown.

Supplementary Table 7

c-MYC and chromosome centromere 8 copy number in radiogenic and sporadic breast cancer

Sample	Mean c-MYC copy number	Mean chromosome 8 copy number	c-MYC: chromosome 8 copy number ratio[A]	% of nuclei c-MYC copy number ≥ 3	% of nuclei chromosome 8 copy number ≥ 3	% of tumour nuclei in each sample[B]
SPO2	3.08	1.62	1.90	42	0	80
SPO5	2.05	2.12	0.97	11	14	55
SPO6	1.94	1.88	1.03	7	4	70
SPO7	2.84	2.84	1.00	48	44	70
SPO10	2.02	2.16	0.94	5	13	85
SPO11	3.22	2.94	1.10	38	34	75
SPO13	2.04	2.06	0.99	10	8	90
SPO15	1.91	1.94	0.98	1	3	45
SPO17	2.95	3.19	0.92	40	48	80
SPO18	2.20	2.26	0.97	24	27	50
SPO21	2.58	2.34	1.10	28	31	70
SPO22	2.00	1.92	1.04	9	7	50
SPO24	2.32	2.03	1.14	25	15	65
SPO26	2.59	2.46	1.05	52	46	75
SPO27	2.28	2.09	1.09	25	16	55
SPO28	2.62	2.04	1.28	48	10	35
SPO29	2.94	2.87	1.02	57	50	80
SPO30	2.00	1.7	1.18	12	8	45
SPO31	2.05	2.11	0.97	12	16	20
SPO32	2.07	2.01	1.03	10	16	50
RAD1	3.38	3.00	1.13	56	49	75
RAD2	2.02	2.01	1.00	4	7	85
RAD3	2.53	2.19	1.16	51	36	90
RAD4	2.00	1.98	1.01	3	2	30
RAD5	2.95	2.44	1.21	45	35	90
RAD8	3.87	3.10	1.25	77	56	80
RAD9	4.01	1.99	2.02	59	8	90
RAD10	5.28	2.11	2.50	47	15	65
RAD17	3.22	2.91	1.11	42	38	85

[A] For each sample mean *c-MYC* and chromosome 8 copy number was calculated. The ratio between these values was calculated by dividing mean *c-MYC* copy number score by mean centromere 8 copy number score.

[B] The estimated percentage of tumour nuclei counted in each sample was determined following histopathalogical examination of the paraffin embedded tissue samples used for nuclei extraction.

Supplementary Table 8

***c-MYC* copy number and c-MYC expression histoscores of sporadic and radiogenic breast cancer cohorts analysed by FISH.**

Sample	c-MYC Score	Histoscore
SPO2	3.08	147.6
SPO5	2.05	0
SPO6	1.94	2
SPO7	2.84	0
SPO10	2.02	0
SPO11	3.22	12.2
SPO13	2.04	6.6
SPO15	1.91	0
SPO17	2.95	47
SPO18	2.20	0
SPO21	2.58	29.8
SPO22	2.00	0
SPO24	2.32	0
SPO26	2.59	0
SPO27	2.28	27.8
SPO28	2.62	0
SPO29	2.94	17.2
SPO30	2.00	72.2
SPO31	2.05	64.2
SPO32	2.07	0
RAD1	3.38	156.2
RAD2	2.02	0
RAD3	2.53	35.2
RAD4	2.00	131.5
RAD5	2.95	52.6
RAD8	3.87	0
RAD9	4.01	70.4
RAD10	5.28	63.4
RAD17	3.22	0
